# Hydronephrosis Classifications: Has UTD Overtaken APD and SFU? A Worldwide Survey

**DOI:** 10.3389/fped.2021.646517

**Published:** 2021-04-12

**Authors:** Santiago Vallasciani, Anna Bujons Tur, John Gatti, Marcos Machado, Christopher S. Cooper, Marie Klaire Farrugia, Huixia Zhou, Mohammed El Anbari, Pedro-José Lopez

**Affiliations:** ^1^Division of Urology, Department of Surgery, Sidra Medicine, Doha, Qatar; ^2^Division of Pediatric Urology, Puigvert Foundation, Barcelona, Spain; ^3^Division of Pediatric Urology, Children's Mercy Hospital, Kansas City, MO, United States; ^4^Division of Pediatric Urology, University of São Paulo, São Paulo, Brazil; ^5^Department of Urology, University of Iowa Hospitals and Clinics, Iowa City, IA, United States; ^6^Division of Pediatric Urology, Chelsea and Westminster Hospital NHS Foundation Trust, London, United Kingdom; ^7^Department of Pediatric Urology, Bayi Children's Hospital, Affiliated of the Seventh Medical Center of PLA General Hospital, Beijing, China; ^8^Division of Clinical Informatics, Sidra Medicine, Doha, Qatar; ^9^Hospital Exequiel Gonzalez Cortes & Clinica Alemana, Santiago, Chile; ^10^University of Chile, Santiago, Chile

**Keywords:** hydronephrosis, classification, survey, pediatric urology, ultrasound, pediatric radiology

## Abstract

**Objective:** To collect baseline information on the ultrasonographic reporting preferences.

**Method:** A 13-multiple choice questionnaire was designed and distributed worldwide among pediatric urologists, pediatric surgeons, and urologists. The statistical analysis of the survey data consisted of 3 steps: a univariate analysis, a bivariate and a multivariate analysis.

**Results:** Three hundred eighty participants responded from all the continents. The bivariate analysis showed the significant differences in the geographical area, the years of experience and the volume of cases. Most of the physicians prefer the SFU and APD systems because of familiarity and simplicity (37 and 34%, respectively). Respondents noted that their imaging providers most often report findings utilizing the mild-moderate-severe system or the APD measurements (28 and 39%, respectively) except for North America (SFU in 50%). Multivariate analysis did not provide significant differences.

**Conclusion:** Our study evaluates the opinions regarding the various pediatric hydronephrosis classification systems from a large number of specialists and demonstrates that there is no single preferred grading system. The greatest reported shortcoming of all the systems was the lack of universal utilization. The observations taken from this study may serve as basis for the construction of a common worldwide system. As APD and SFU are the preferred systems and the UTD a newer combination of both, it is possible that with time, UTD may become the universal language for reporting hydronephrosis. This time, based on the result of this survey, seems not arrived yet.

## Introduction

Ultrasound reports serve as an instrument to communicate anatomic findings to health care providers permitting the patient's physician to make therapeutic decisions and counsel families. In the specific case of hydronephrosis, the report can be generated from the maternal-fetal specialist in the prenatal period or the pediatric radiologist postnatally. To communicate the results of the ultrasound study reliably and accurately, several classifications have been developed. Initially the anterior-posterior diameter of the renal pelvis (APD) was developed. Subsequently, additional systems that included other anatomical details regarding the calyces, renal parenchyma, ureters, and/or bladder were developed. These classification systems included the Society of Fetal Urology (SFU), Onen, UTD (Urinary Tract Dilatation), and European Society of Pediatric Radiology system (ESPR).

To date, there is no clear consensus on which of these systems offer better categorization of the dilatations, the best inter/intra-rater reliability, or the best prognostic value at the time of its assessment in cases of suspected or diagnosed urinary tract obstruction or vesicoureteric reflux. Even among pediatric urologists and surgeons, the individuals who will utilize these reports to make therapeutic and surgical decisions, no apparent consensus exists on which system is preferable. To advance communication and subsequent research in this area, a clear consensus among pediatric urologists regarding the preferable system for categorization and reporting of hydronephrosis is needed.

We hypothesize that there is no single preferred hydronephrosis grading system among pediatric surgeons and urologists. The aim of this study was to collect baseline information on the ultrasonographic reporting preferences among pediatric urologists and surgeons evaluating hydronephrosis and correlate it with the reporting system utilized in their localities.

## Materials and Methods

A 13-multiple choice questionnaire was designed by the authors (Appendix 1 in Supplementary Material). It was comprised of 4 questions on surgical specialty and type of practice, 4 on classification preferences, 3 related to communication with report providers, and 2 on future perspectives (Appendix 1 in Supplementary Material).

Institutional Review Board of the Institution of the first author waived the review by them as considered not a requirement for the present research. The participation of the responders was voluntary and considered as consent. The responders were also able to decide whether to provide their names and email contact or remain anonymous.

From November 2018 to February 2019, the questionnaire was accessible online through GoogleForm(R) platform and publicized through mailing lists (peds-urology@lists.it.uab.edu, novo-uroped@googlegroups.com, European Society for Pediatric Urology roster members database) and social media groups (Sociedad Iberoamericana de Urologia Pediatrica, Argentinian, Chilean, and Brazilian Society for Pediatric Urology). The Society for Fetal Urology advertised it through their members. Colleagues in China had an alternative link to the same survey through SurveyMonkey(R). Duplicate respondents were avoided as these survey platforms identify the respondent before allowing them to submit the survey. A secondary assessment of potential duplicated responses was performed manually by the authors reviewing case by case the answers.

The statistical analysis of the survey data consists of 3 steps: a univariate analysis by providing the frequencies and representing graphically each variable alone; a bivariate analysis by measuring dependence of each variable from a first group with each variable from a second group, this is done using a G-test which is more general than a chi-square test; and a multivariate analysis using Multiple Correspondence Analysis (MCA) to form groups of the surveyed people depending on their answers to all the questions. All the statistical analyses are performed using the R statistical software version 3.5.0.

## Results

**Figure 1:**
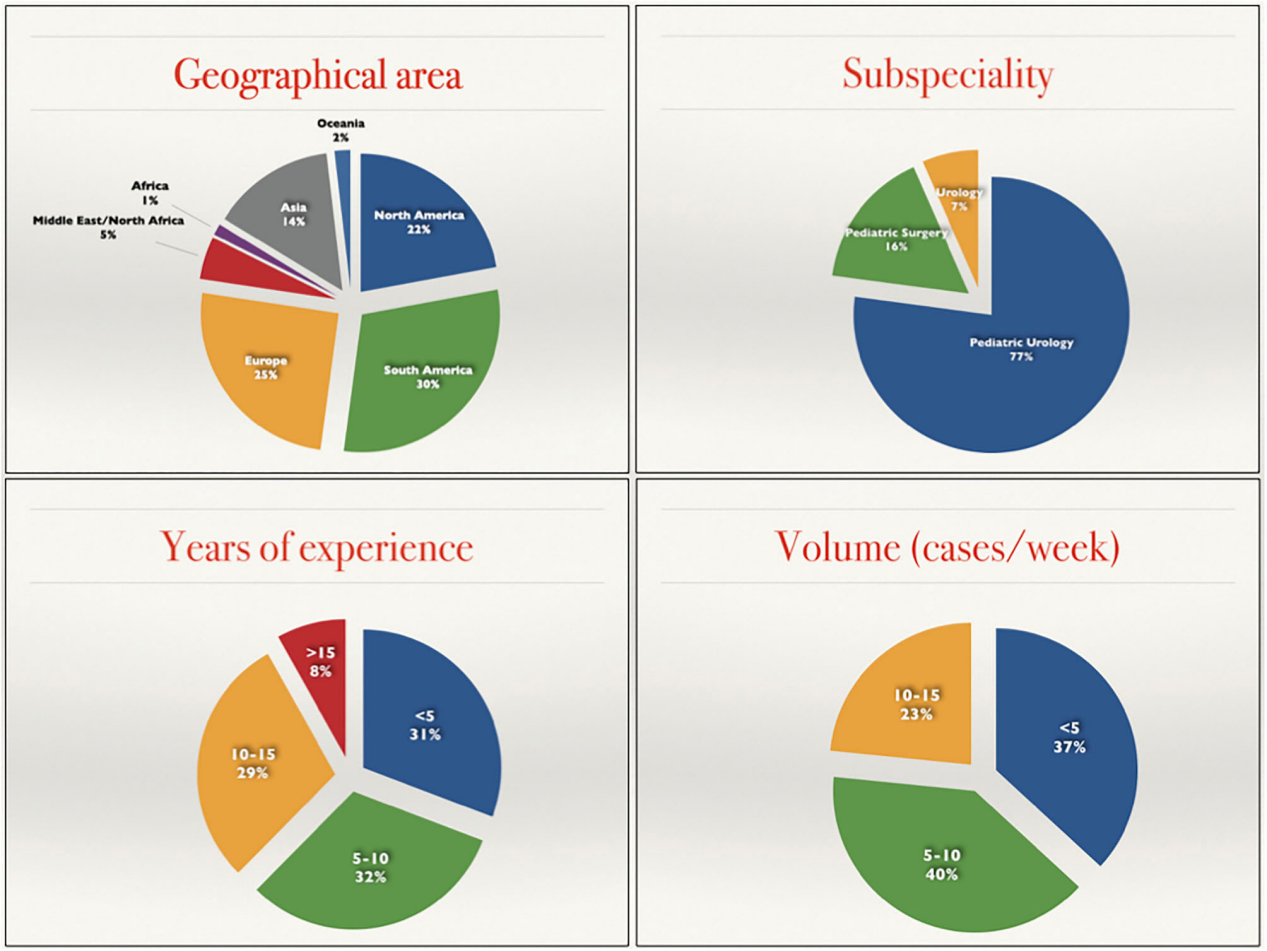
Demographic details of the 380 participants to the study.

**Figure 2:**
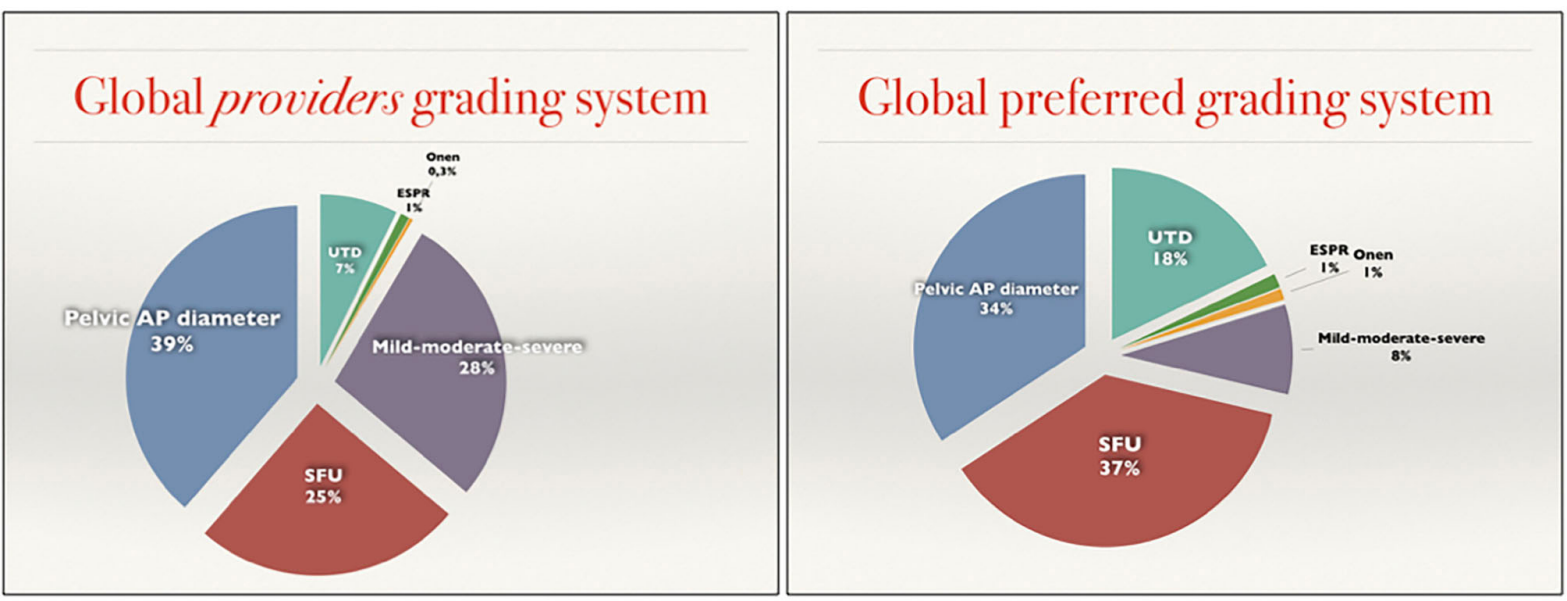
Graphic representation of the differences between Grading systems provided and Grading systems preferred.

Three hundred and eighty physicians participated to the questionnaire. The univariate analysis results are depicted in the Figures 1, 2.

Globally, the two most preferred systems were the SFU system and the renal Pelvic AP diameter with 37 and 34%, respectively (140/380 and 129/380). The more recently developed UTD system ranked third in terms of overall preference with 18% choosing it as their preference. A minority of participants (8%) choose the mild-moderate-severe system and only 1% chose the Onen or the ESPR system (Figure 2, right).

The classification systems most utilized by providers was based on an open question (question 11) that permitted the participant to choose more than one classification system. Globally there were 601 responses to this question, averaging nearly 2 study systems per respondent. This resulted in an increase of the popularity of the mild-moderate-severe (28%) and a net reduction on SFU and UTD (25 and 7). The Pelvic AP diameter was slightly increased (39%) while the Onen and ESPR remained uncommon (0.3 and 1%) (Figure 2, left).

The bivariate analysis (Table 1) showed significant differences in the type of responses. Three main variables affected these differences: The geographical area for favorite classification system, communication with providers, system used by providers and attempt to build a common system, the years of experience attempting to build a common system and willingness to change the preferred system, and the volume of cases per provider.

**Table 1:** *P*-values corresponding to a G-test of independence between the variables Q5, Q7, Q8, …Q13 and the demographic variables Q1, …, Q4.

	**Q1. What is your subspecialty?**	**Q2. What is your geographical area?**	**Q3. Years of experience in Pediatric Urology**	**Q4. How many cases of hydronephrosis you manage in a typical week?**
Q5. When you deal with a case of hydronephrosis, which is your favorite classification system?	0.05201	**0.00000007**	0.3287	**1.955e-06**
Q7. Why do you prefer the system you use? (you can choose more than one)	0.4818	0.07535	0.7218	0.2789
Q8. What are the shortcomings of the system you use? (you can choose more than one)	0.9644	0.3354	0.6167	0.6515
Q9. Do you have direct communication with your radiology report providers?	0.3372	**0.001802**	0.1221	0.1835
Q10. If yes, how often?	0.4125	**0.00004**	0.938	0.257
Q11. Which is the most frequently used classification system you see in your practice (the one most used by your providers)? (you can choose more than one)	0.9974	**0.0000005**	0.2386	**0.0004758**
Q12. Did you attempt to build a common language for description of hydronephrosis among your own team?	0.1706	**0.001403**	**0.008423**	0.4134
Q13. Are you available to change your preference in case the majority of Pediatric Urologist prefers another grading system?	0.586	0.1987	**0.0403**	0.6104

*We see that the responses to question Q5 depend on the demographic variables Q2 and Q4. Q9 is dependent on Q2. Q10 depends on Q2. The answers to Q11 depend on Q2 and Q4 while the answers to Q12 are associated with Q2 and Q3. Finally, the categories taken by Q13 depends on the categories taken by Q3. Bolded P values < 0.005.*.

**Figure 3:**
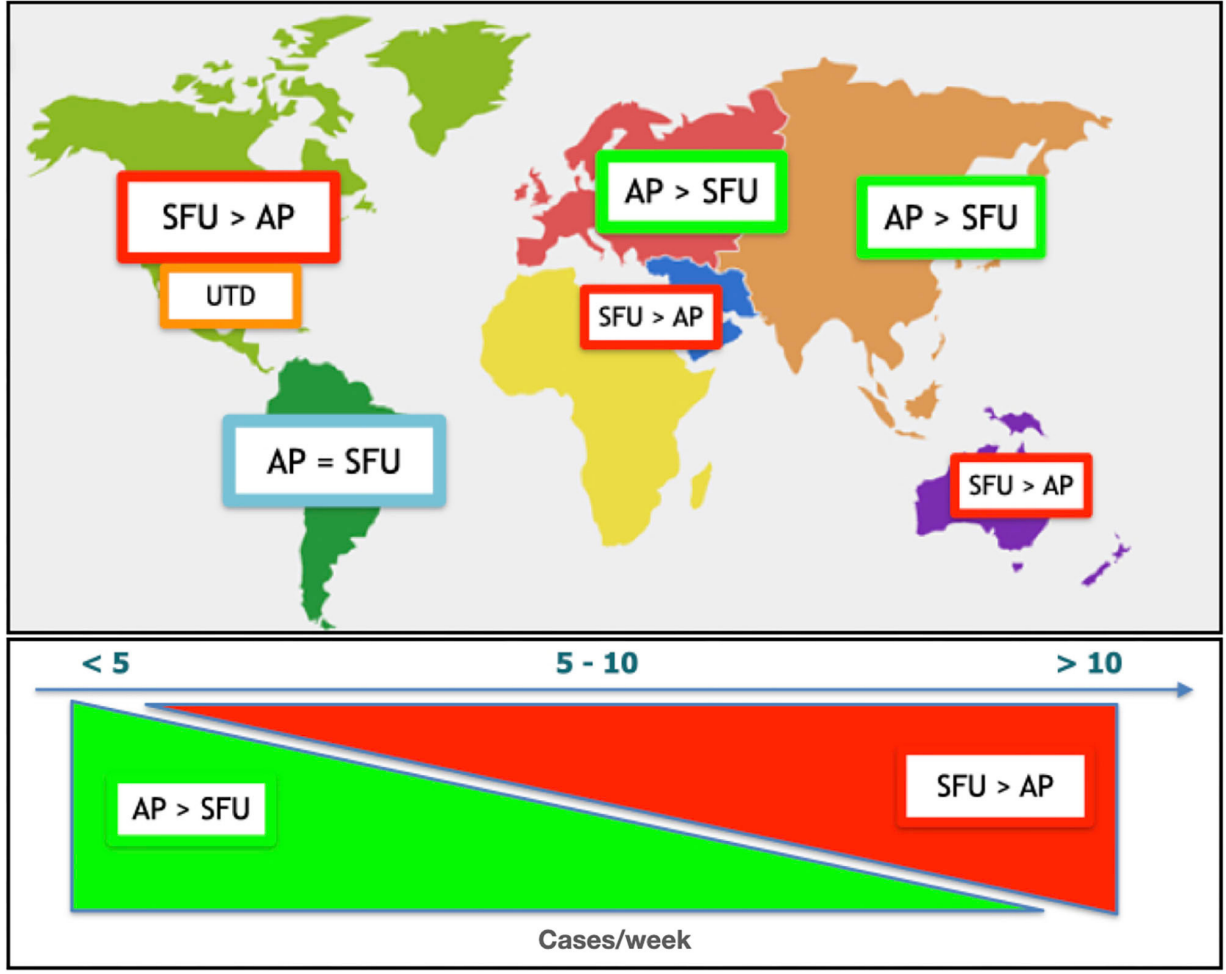
Difference of preferences based on geographical provenience **(Top)** and cases per week volume **(Bottom)**.

There were significant differences in preferred grading system related to the geographical area (Supplementary Table 1). Asia, Europe and Oceania prefer the Pelvic AP diameter (47, 45, and 57%) whereas Middle East/North Africa and North America prefer the SFU system (63 and 59%). South America did not show a marked difference among the Pelvic AP diameter and SFU (38 and 30%). Within geographic areas, there were major differences in communication with providers. The majority of the participants described direct communication to varying degrees (Supplementary Table 2). In Europe, North America and Oceania this is more common than in the rest of the world. The frequency of this contact is also different with the higher frequency in Europe and Oceana. The grading system used by providers also varied geographically (Supplementary Table 3). In all the regions except North America the most utilized system by the providers is the Pelvic AP diameter. In North America, the SFU system is the most frequently used. In most areas, there was an attempt to build a common system, but more common in Europe, South America, and Oceania (Figure 3 and Supplementary Table 4).

Years of experience was associated with an increased attempt to build a common system (Supplementary Table 5). The willingness to change the preferred system (91%, range 88–98) revealed that those who most willing are the group with middle experience (10–15 years) (Supplementary Table 6).

The volume of cases also had an impact on the preferred grading system (Supplementary Table 7). SFU and UTD preference grew with increasing patient volume. In contrast, the low volume responders preferred the Pelvic AP diameter system (Figure 3). The system used by providers (Supplementary Table 8) was similar to the preferred grading system. Higher volumes correlated with preference for SFU and UTD and lower volumes with the mild-moderate-severe system.

The multivariate analysis revealed no statistically significant correlations between all the variables studied (two-dimensional correspondence analysis plot of the questionnaire data using the package ade4 in R with data points labeled by continents is available in the complementary documents of this manuscript).

The participants were able to express their opinions regarding the utility of each system by grading it from “very useful” to “useless” (question 6). This was also an open question permitting multiple responses. Scores were given according to the number of responses in each category except for “not known” which was not scored. In order to assign a numeric value to this answer, each category had a weighted multiplying factor as shown in Table 2. The highest scores for utility were obtained by the Pelvic AP diameter and the SFU systems. The systems categorized as “not known” by most of the participants were the ESPR and the Onen (112 and 127, respectively).

**Table 2:** Opinion of the responders of each system.

	**Very useful (x4)**	**Somewhat useful (x3)**	**Minimally useful (x2)**	**Useless (x1)**	**SCORE**	**Not known**
Mild-moderate-severe system	46 (184)	125 (375)	129 (258)	53	**870**	5
Pelvic AP Diameter measurements	184 (736)	137 (411)	31 (62)	4	**1,213**	2
Society of Fetal Urology system (1)	191 (764)	125 (375)	24 (48)	2	**1,189**	8
ESPR Pediatric Uroradiology Working Group grading (2)	29 (116)	138 (276)	63 (126)	10	**528**	**112**
Onen grading system (3)	22 (88)	120 (360)	64 (128)	17	**593**	**127**
Urinary Tract Dilation (UTD) classification system (4)	113 (452)	142 (426)	51 (102)	4	**984**	**44**

*Bolded values of higher significance*.

Participants were invited to express their opinion regarding the strengths of their preferred systems. Points of strength for mild-moderate-severe, Pelvic AP diameter, and SFU systems was “Familiarity” and “Simplicity.” In addition, “Good prognostic value” was a strength reported for the SFU and UTD systems. The most frequent shortcomings noted were principally that the system was “Not used universally.”

## Discussion

Prenatal and postnatal hydronephrosis is a very common condition affecting approximately 1% of pregnancies. In many countries/areas the role of pre and postnatal counseling and care for hydronephrosis is provided by Pediatric Urologist or by either Pediatric Surgeons or Adult Urologists dedicated to pediatric patients. The goal of a common and objective language in the description of the degree and characteristics of hydronephrosis along with prognostic clinical correlation has been attempted since the wide use of ultrasound as first line investigation in both the prenatal and postnatal period. Dhillon et al. published a detailed report correlating the degree of dilatation with the clinical outcome in terms of need for surgical intervention (5). Although many experienced physicians prefer to independently assess the radiological images rather than rely on reports, the images are not always available adding delays in management decision and timing of intervention.

Grading systems have evolved in complexity over time beginning with the simpler, classic “mild-moderate-severe” system (6) and the anterior-posterior diameter (7). In 1993 the Society for Fetal Urology proposed the SFU classification system for postnatal hydronephrosis (1), followed by the European Society for Pediatric Radiology which proposed its modified system by adding the anterior-posterior diameter (2). In 2007, Onen presented his individual experience with a modified system aimed to better stratify the ultrasound characteristics of the hydronephrotic kidney and its clinical significance (3). Finally, a consensus among several societies of Pediatric Urology, Nephrology and Radiology was accomplished in 2014 and resulted in the Urinary Tract Dilatation system (4). This system introduced additional characteristics of the urinary tract not considered in the previous systems including ureteric dilation and bladder abnormalities and can be considered an integration of the SFU and anterior-posterior diameter systems.

The evolution of classification systems has attempted to improve prognostic ability by combining additional sonographic findings. The use of multiple different classification systems makes communication and translation of research findings difficult. Over the last 30 years, multiple studies have been done evaluating the strengths and challenges of the various classification systems. Considering multiple specialities, Zanetta et al. (8) demonstrated lack of agreement within different specialities involved in the management of hydronephrosis both in grading system and management. Our study uniquely evaluates opinions regarding the various pediatric hydronephrosis classification systems from a large number of surgical specialists from throughout the world.

Our study supports our hypothesis that that there is no single preferred hydronephrosis grading system among pediatric surgeons and urologists. The geographical differences were subtle in some areas while particularly marked in others. This may reflect agreement between regional societies or presence of leadership opinions that influence preferences toward a particular system.

Our study is not without limitation. Although we had 380 respondents, it is not known how representative this group is of the global census of physicians that manage fetal and pediatric hydronephrosis. Currently there is no estimation of the number of physicians (pediatric urologists, pediatric surgeons) practicing worldwide. Based on the organization with the highest number of physicians dedicated to Pediatric Urology, the European Society for Pediatric Urology whose roster is of 790 members from different areas of the world (www.espu.org website) plus another 450 certified by SPU and SFU, it can be hypothesized that the number of respondents to the present survey represents a significant portion of the physicians managing cases of children with hydronephrosis. The utilization of multiple sources of engagement and repetition of the invitations was a strategy to enhance inclusion and representation as recommended by Ponto in the paper on surveys as a research tool (9).

The heterogeneity in “years of experience,” “subspeciality,” and “geographic area” are also limitations of the study. The lack of an overriding organization for physicians treating hydronephrosis necessitated broad solicitation of voluntary participation by physicians of differring backgrounds. Another limitation is that the opinions were expressed anonymously [although 284/380 (73%) participants voluntarily disclosed their identity] making it impossible to assess the validity of all responses.

## Conclusion

The present survey demonstrates that there is no single preferred hydronephrosis grading system among pediatric surgeons and urologists. Despite a clear favorite, even with regional variations, most of the physicians charged with the management of pediatric hydronephrosis prefer the SFU and APD systems because of familiarity and simplicity with these systems (37 and 34%, respectively). Respondents noted that their imaging providers most often report findings utilizing the mild-moderate-severe system or the APD measurements (28 and 39%, respectively) except for North America where the SFU system is more seen (50%). The greatest reported shortcoming of all the systems was the lack of universal utilization. Nearly all respondents were optimistic that if a consensus regarding a classification system was determined, they would be able to have this new system implemented at their institution. The observations taken from this study may serve as basis for the construction of a common worldwide system among physicians managing hydronephrosis and imaging providers. As APD and SFU are the preferred systems and the UTD a newer combination of both, it is possible that with time, UTD may become the universal language for reporting hydronephrosis. The result of this survey, however, shows that this time has not come yet.

## Data Availability Statement

The raw data supporting the conclusions of this article will be made available by the authors, without undue reservation.

## Author Contributions

SV, AB, JG, MM, CC, and P-JL: conception and design. SV, AB, JG, MM, CC, MF, HZ, and P-JL: acquisition of data. SV and ME: analysis and interpretation of data. SV, ME, JG, and CC: drafting of the manuscript. JG, CC, MF, and P-JL: critical revision of the manuscript for important intellectual content. ME: statistical analysis. All authors contributed to the article and approved the submitted version.

## Conflict of Interest

The authors declare that the research was conducted in the absence of any commercial or financial relationships that could be construed as a potential conflict of interest.

## Supplementary Material

Supplementary Figure 1 Two-dimensional correspondence analysis plot of the questionnaire data using the package ade4 in R. The data points are labeled by continents.

Supplementary Table 1 Preferred system by geographical area.

Supplementary Table 2 Communication with providers by geographical area.

Supplementary Table 3 System by providers by geographical area.

Supplementary Table 4 Attempt to build a common system by geographical area.

Supplementary Table 5 Attempt to build a common system by years of experience.

Supplementary Table 6 Availability to change the preferred system by years of experience.

Supplementary Table 7 Preferred system by number of cases seen by week.

Supplementary Table 8 System by providers by number of cases seen by week.
