# Supplementary figures and images for: Hydronephrosis Classifications: Has UTD Overtaken APD and SFU? A Worldwide Survey
Source: Front Pediatr. 2021 Apr 12;9:646517. doi: 10.3389/fped.2021.646517 (PMC8072019; doi:10.3389/fped.2021.646517)

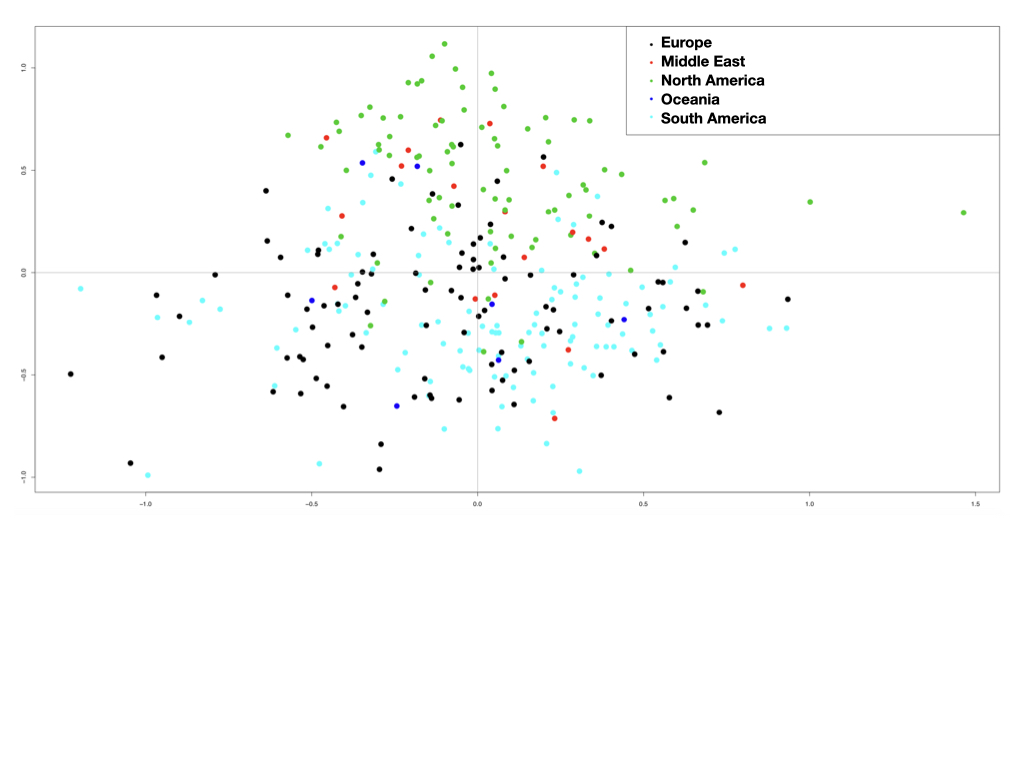

Supplement: Supplementary Figure 1 — Two-dimensional correspondence analysis plot of the questionnaire data using the package ade4 in R. The data points are labeled by continents. [file Image_1.JPEG]
