# Supplementary material for: Hydronephrosis Classifications: Has UTD Overtaken APD and SFU? A Worldwide Survey
Source: Front Pediatr. 2021 Apr 12;9:646517. doi: 10.3389/fped.2021.646517 (PMC8072019; doi:10.3389/fped.2021.646517)
Supplement: Supplementary Table 1 — Preferred system by geographical area. [file Data_Sheet_1.docx]

SUPPLEMENTARY MATERIAL

ANNEX 1: survey submitted to the participants

1. Subspeciality
   - - - Pediatric urology
       - Pediatric surgery
       - Urology
       - other (specify)
2. Geographical area
   - - - North America
       - South America
       - Europe
       - Middle East North Africa
       - Africa
       - Asia
       - Oceania
3. Years of experience in Pediatric Urology
   - - - less than 5
       - 5 to 10
       - 10-15
       - over 15
4. How many cases of hydronephrosis you manage in a typical week?
   - - - more than 10
       - 5-10
       - less than 5
5. When you deal with a case of hydronephrosis, which is your favorite classification system?
   - - - Mild-moderate-severe system
       - Pelvic AP Diameter measurements
       - Society of Fetal Urology system (Pediatr Radiol 1993, 23:478–480)
       - ESPR Pediatric Uroradiology Working Group grading (Pediatr Radiol 2008, 38:138-145)
       - Onen grading system (J Pediatr Urol 2007, 3:200-205)
       - Urinary Tract Dilation (UTD) classification system (J Ped Urol 2014;10:982-99).

6) Can you please score the clinical usefulness of the following classification systems based on your opinion/previous experiences/knowledge?

|  | Very useful | Somewhat useful | Minimally useful | Useless | Not known |
| --- | --- | --- | --- | --- | --- |
| Mild-moderate-severe system |  |  |  |  |  |
| Pelvic AP Diameter measurements |  |  |  |  |  |
| Society of Fetal Urology system (Pediatr Radiol 1993, 23:478–480) |  |  |  |  |  |
| ESPR Pediatric Uroradiology Working Group grading (Pediatr Radiol 2008, 38:138-145) |  |  |  |  |  |
| Onen grading system (J Pediatr Urol 2007, 3:200-205) |  |  |  |  |  |
| Urinary Tract Dilation (UTD) classification system (J Ped Urol 2014;10:982-99). |  |  |  |  |  |

7) Why do you prefer the system you use?

- - - - Familiarity
      - Simplicity
      - Widely accepted
      - Good prognostic value

8) What are the shortcomings of the system you use?

- - - - Unfamiliarity
      - Not used universally
      - Complicated
      - Lack of prognostic value

9) Do you have direct communication with your radiology report providers?

- - - - yes
      - no

10) If yes, how often?

- - - - more than once a week
      - once a month
      - rarely
      - n/a (click if you reply no to the previous question)

11) Which is the most frequently used classification system you see in your practice (the one most used by your providers)? (you can choose more than one)

- - - - Mild-moderate-severe system
      - Pelvic AP diameter system
      - SFU system
      - ESPR grading
      - Onen grading system
      - UTD classification system

12) Did you attempt to build a common language for description of hydronephrosis among your own team?

- - - - yes
      - no

13) Are you available to change your preference in case the majority of Pediatric Urologist prefers another grading system?

- - - - yes
      - no

ADDITIONAL TABLES

Table 1: **Preferred system by Geographical area**

Asia Europe M.East - N.Africa North America Oceania South America

ESPR 3.63 2.08 0 0 0 0.87

Mild_Moderate_Severe 18.18 2.08 5.26 10.71 0 7.01

Onen 0 3.12 0 0 0 0.87

Pelvic_AP_Diameter **47.27** **45.83** 26.31 5.95 **57.14** **38.59**

SFU 18.18 32.29 **63.15** **59.52** 28.57 **30.70**

UTD 12.72 14.58 5.26 23.80 14.28 21.92

*Total 100 100 100 100 100 100*

Table 2: **Communication with providers by Geographical area**

Asia Europe M.East - N.Africa North America Oceania South America

No 30.90 12.50 26.31 7.14 0 22.80

Yes 69.09 87.50 73.68 92.85 100 77.19

*Total 100 100 100 100 100 100*

More than once a week 30.76 70.37 42.85 53.65 71.42 51.61

Once a month 30.76 25.92 35.71 36.58 28.57 22.58

Rarely 38.46 3.70 21.42 9.75 0 25.80

*Total 100 100 100 100 100 100*

Table 3: **System by providers by Geographical area**

Asia Europe M.East - N.Africa North America Oceania South America

ESPR 0 1.88 0 0 0 0

Mild-moderate-severe 23.07 9.43 44.44 30.43 33.33 32.65

Onen 0 3.77 0 0 0 0

Pelvic AP diameter 56.41 54.71 33.33 2.17 66.66 42.85

SFU 12.82 28.30 22.22 50.00 0 22.44

UTD 7.69 1.88 0 17.39 0 2.04

*Total 100 100 100 100 100 100*

Table 4: **Attempt to build a common system by Geographical area**

Asia Europe M.East - N.Africa North America Oceania South America

No 32.72 12.50 31.57 32.14 0 14.91

Yes 67.27 87.50 68.42 67.85 100 85.08

*Total 100 100 100 100 100 100*

Table 5: **Attempt to build a common system by Years of experience**

Over 15 10 to 15 5 to 10 Less than 5

No 15.15 17.39 34.24 26.38

Yes 84.84 82.60 65.75 73.61

*Total 100 100 100 100*

Table 6: **Availability to change the preferred system by Years of experience**

Over 15 10 to 15 5 to 10 Less than 5

No 11.51 1.44 9.58 8.33

Yes 88.48 98.55 90.41 91.66

*Total 100 100 100 100*

Table 7: **Preferred system by Number of cases seen by week**

Less than 5 5_to_10 More than 10

ESPR 2.94 0 1.12

Mild_Moderate_Severe 6.61 8.66 8.98

Onen 2.20 0 1.12

Pelvic_AP_Diameter 48.52 32.66 14.60

SFU 27.20 42.00 44.94

UTD 12.50 16.66 29.21

*Total 100 100 100*

Table 8: **System by providers by Number of cases seen by week**

Less than 5 5_to_10 More than 10

ESPR 0 1.28 0

Mild-moderate-severe 26.58 25.64 19.04

Onen 0 1.28 2.38

Pelvic AP diameter 37.97 44.87 30.95

SFU 27.84 26.92 30.95

UTD 7.59 0 16.66

*Total 100 100 100*
